# A Hybrid Approach for Short-Term Forecasting of Wind Speed

**DOI:** 10.1155/2013/548370

**Published:** 2013-12-24

**Authors:** Sivanagaraja Tatinati, Kalyana C. Veluvolu

**Affiliations:** School of Electronics Engineering, College of IT Engineering, Kyungpook National University, Daegu, Republic of Korea

## Abstract

We propose a hybrid method for forecasting the wind speed. The wind speed data is first decomposed into intrinsic mode functions (IMFs) with empirical mode decomposition. Based on the partial autocorrelation factor of the individual IMFs, adaptive methods are then employed for the prediction of IMFs. Least squares-support vector machines are employed for IMFs with weak correlation factor, and autoregressive model with Kalman filter is employed for IMFs with high correlation factor. Multistep prediction with the proposed hybrid method resulted in improved forecasting. Results with wind speed data show that the proposed method provides better forecasting compared to the existing methods.

## 1. Introduction

Exponential increase in energy consumption globally is leading to rapid depletion of existing fossil fuel resources [1]. This impending scarcity has led the power industry to explore renewable energy sources such as wind, solar, and tidal energies [2, 3]. Renewable energy resources attract more attention owing to their pollution free energy generation capabilities. Wind as a potential source for generation of electricity on a large scale has been receiving much attention recently. In China alone, the growth rate of wind farms was reported as 114% in 2009 with the total wind generation capacity of 25805.3 MW [4]. However, stable production of electricity from wind power is a quite arduous task due to the uncertainty and intermittency of wind speed. The increasing importance of wind energy, affected by variations in wind speed, necessitates accurate forecasting of wind speed.

In recent past, significant amount of research has been focused on forecasting the wind speed. However, due to the properties of wind speed such as nonstationarity, high fluctuations, and irregularity, accurate forecasting becomes a challenge. Generally, forecasting of wind speed is classified into two types: (1) short-term forecasting and (2) long-term forecasting. Short-term forecasting of wind speed affects grid reliability and market-based ancillary service costs [5, 6], whereas long-term forecasting provides an idea about a particular site location [7]. The prediction models proposed in the recent past for wind speed prediction are categorized as physical models, time series statistical models, and knowledge-based methods. Each model has its own advantages and disadvantages. Physical models such as Markov models [8] require information regarding the temperature and climatic conditions to build the models. In time series statistical modeling, various techniques such as autoregressive moving average (ARMA) [9, 10], autoregressive integrated moving average (ARIMA) [11], Kalman filter [12], model-based approaches [13–15], and Particle swarm optimization [16] are employed for prediction. Knowledge-based methods have been the widely adapted techniques for wind speed forecasting especially artificial neural networks (ANN) [17, 18], radial bias function [19], fuzzy logic [20], and support vector machines [7].

Recently, hybrid methods based on *divide and conquer* principle are proposed for accurate forecasting of wind speed [21–23]. In these methods, wind speed data is decomposed into independent components. Later, each component is predicted with adaptive algorithms such as ARMA, SVM, or ANN. In [23], empirical mode of decomposition (EMD) was employed to decompose the signal into intrinsic mode functions (IMFs), and then ARMA model with fixed coefficients was employed to forecast the IMFs individually (EMD-ARMA). Further, SVM and ANN are employed to predict the IMFs in [22] and [21], respectively, to improve the forecasting performance. Different IMFs obtained from EMD posses different frequency bands and characteristics. For instance, IMF-n in the lowest frequency band represents the central tendency of data, and IMF-1 is the highest frequency band and it mainly contains a large quantity of noisy signals. Although regression models are effective for time series prediction, owing to the highly nonstationary characteristics of few IMFs (high frequency IMFs), the prediction with regression models is not effective. On the other hand, the performance of machine learning techniques (SVM, ANN) for low frequency components may hamper due to the over fitting of data.

To overcome the limitations, in this paper, we propose a new hybrid approach for multistep prediction of the IMFs. Instead of employing a single adaptive algorithm for predicting the IMFs, we employed the combination of LS-SVM and AR for prediction of IMFs. Based on the partial autocorrelation factor (PACF) and the frequency characteristics of IMFs, the adaptive prediction algorithm will be identified. In the proposed hybrid approach, for high frequency IMFs (weak correlation factor), LS-SVM is employed, and for low frequency IMFs (high correlation factor) AR model with Kalman filter is employed. Results show that the proposed hybrid approach provides better forecasting performance compared to the existing methods.

The paper is organized as follows. In Section 2, brief description of LS-SVM, AR model with Kalman filter, and the proposed hybrid approach is discussed.  Section 3 provides wind speed data collection procedure, obtained results, and implications.  Section 4 concludes the paper.

## 2. Methodology

In this section, we first discuss the formulation of all the methods (EMD, AR model with Kalman filter, and LS-SVM), followed by the proposed hybrid approach.

### 2.1. Empirical Mode Decomposition (EMD) [24]

**Figure 1:**
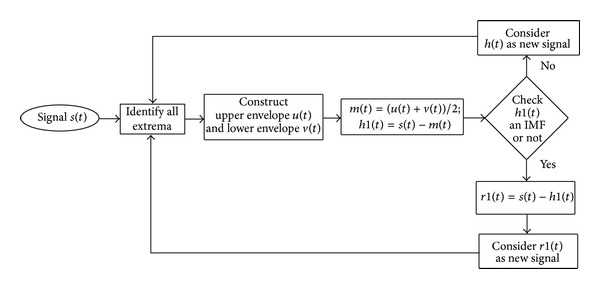
Flowchart of EMD.

EMD has been a widely accepted method for decomposition of nonlinear and nonstationary signals. The basic idea of EMD is to identify the steady-state intrinsic oscillatory modes by employing Hung-Hilbert transform. The detailed procedure for EMD decomposition technique is well documented; for details see [24, 25]. In Figure 1, the flowchart representation of EMD process is shown.

The process of EMD to decompose the signal *s*(*t*) is as follows.

- Step 1: initially, all extrema of *s*(*t*) will be identified by a cubic spline.
- Step 2: the mean value (*m*(*t*)) of upper envelope (*u*(*t*)) and lower envelope (*v*(*t*)) is calculated, *m*(*t*) = ((*u*(*t*) + *v*(*t*))/2), and subtracted from *s*(*t*) to obtain *h*(*t*), *h*1(*t*) = *s*(*t*) − *m*(*t*).
- *h*1(*t*) can be an IMF, if it obeys the following conditions.
  - In the whole data series, the number of extrema and the number of zero crossings in a whole sampled data set must either be equal or differ at most by one.
  - At any point, the mean value of the envelope defined by the local maxima and the envelope defined by local minima is zero.
- Step 3: if *h*1(*t*) does not obey the above conditions, then *h*1(*t*) will be considered as new signal and the same procedure from Step 1 will be followed.
- Step 4: if *h*1(*t*) is an IMF, then residue (*r*1(*t*)) for *h*1(*t*) will be calculated, *r*1(*t*) = *s*(*t*) − *h*1(*t*). Consider *r*1(*t*) as new signal and same procedure from Step 1 will be employed.

### 2.2. Autoregressive Model (AR) with Kalman Filter [26]

AR model is a type of random process which is popular for prediction of various types of natural phenomena. It is also one of the linear prediction methods designed to predict an output of a system based on the previous outputs and the regression coefficients (weights). In this paper, Kalman filter is combined with the AR model to update the weights to enhance the prediction quality.

The state-space model of AR model of order *M* can be given by

*Measurement equation:*

(1) $$\begin{array}{c} s_{t}=w_{t}^{T}s_{t}+\varepsilon_{t}. \end{array}$$

*State equation: *

(2) $$\begin{array}{c} w_{t+1}=w_{t}+\eta_{t}, \end{array}$$

where $w_{t}={\left[\begin{array}{cccc} -w_{1} & -w_{2} & \cdots & -w_{M} \end{array}\right]}^{T}$ represents the weights (time-varying filter coefficients), $s_{t}={\left[\begin{array}{cccc} s_{t-1} & s_{t-2} & \cdots & s_{t-M} \end{array}\right]}^{T}$ represents delayed inputs, and *ε*_*t*_ and *η*_*t*_ are independent white noise process with Gaussian distribution and variance (*σ* ^2^).

The Kalman filter update equations are given by [26]

(3) $$\begin{array}{c} {\hat{w}}_{t+1}={\hat{w}}_{t}+K_{t}\left(s_{t}-x_{t}^{T}{\hat{w}}_{t}\right)\\ e_{t}=s_{t}-s_{t}^{T}{\hat{w}}_{t}\\ K_{t}=\frac{P_{t-1}s_{t}}{s_{t}^{T}P_{t-1}s_{t}+R}\\ P_{t}=\left(I-K_{t}s_{t}^{T}\right)P_{t-1}+Q, \end{array}$$

where *e*_*t*_ represents prediction error, **K**_*t*_ represents Kalman gain vector, **P**_*t*_ represents error covariance matrix, **Q** represents covariance of state noise, and *R* represents covariance of measurement noise.

### 2.3. Least Squares-Support Vector Machines (LS-SVM) [27]

LS-SVM is the least squares version of support vector machine (SVM). In LS-SVM, the regression approximation addresses the problem of estimating a function based on given training data {**s**_*i*_, *y*_*i*_}_*i*=1_^*N*^ with **s**_*i*_ as a *n*-dimensional input vector and *y*_*i*_ as the corresponding output. A brief formulation for LS-SVM is provided here; for more information see [27]. The regression model for LS-SVM can be given in the form:

(4) $$\begin{array}{c} y=\omega^{T}\varphi \left(s\right)+b, \end{array}$$

where *ω* is the weight vector and *b* is the bias.

The optimization problem for the function estimation with LS-SVM is defined as follows:

(5) $$\begin{array}{c} \underset{\omega ,b,e}{\min}J\left(\omega ,\xi \right)=\frac{1}{2}\omega^{T}\omega +C\sum_{i=1}^{N}e_{i}^{2}, \end{array}$$

subject to the constraints *y*_*i*_ = *ω* ^*T*^ *φ*(**s**_*i*_) + *b* + *e*_*i*_; *i* = 1,2,…, *N*, where *C* is a regularization constant and *e*_*i*_ is the estimation error.

The Lagrangian function for the optimization problem can be given as

(6) $$\begin{array}{c} L\left(\omega ,b,e;\alpha \right)=J\left(\omega ,e\right)-\sum_{i=1}^{N}\alpha_{i}\left[\omega^{T}\varphi \left(s_{i}\right)+b+e_{i}-y_{i}\right], \end{array}$$

where *α* = [*α*_1_, *α*_2_,…, *α*_*N*_] represents the Lagrangian multipliers.

The regression model with LS-SVM can be obtained as

(7) $$\begin{array}{c} \hat{y}\left(t+T\right)=\sum_{i=1}^{N}\alpha_{i}K\left(s_{i},s_{k}\right)+b;\;t=N+1,\ldots ,l, \end{array}$$

where *K*(·, ·) represents the Kernel function. RBF Kernel employed in this paper is *K*(**s**, **s**_*i*_) = exp⁡{−||**s**−**s**_*i*_||^2^/*σ* ^2^}.

### 2.4. The Hybrid EMD-LSSVM-AR Model

**Figure 2:**
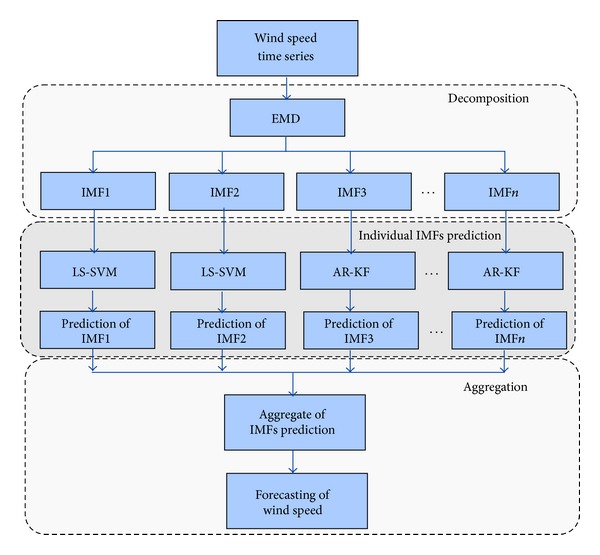
The framework for hybrid EMD-LSSVM-AR model.

The procedure for forecasting the wind speed with the proposed hybrid method comprises three stages as shown in Figure 2. In the first stage, owing to the nonstationary and stochastic characteristics of wind speed time series, the signal will be decomposed into meaningful local time scales by employing EMD [28]. In the second stage, prediction of all the decomposed components will be performed individually with either LS-SVM or AR model. The selection of adaptive algorithm for an IMF is based on the obtained PACF factor and frequency components of the corresponding IMF. LS-SVM is employed for weakly correlated IMFs and AR model for the highly correlated IMFs. In the final stage, the predictions are aggregated to attain the final forecasting result.

## 3. Results and Discussion

In this section, data employed for prediction is described. Following that, the indices were employed to evaluate the forecasting performance. Finally, performance analysis with the existing methods is discussed.

### 3.1. Wind Speed Data

**Figure 3:**
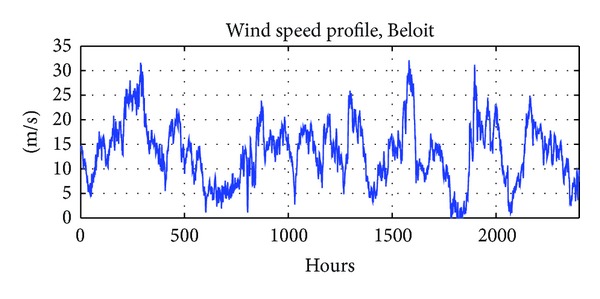
Hourly wind speed profile of Beloit.

Wind data collected from Beloit, Kansas, from a 20-meter anemometer as an integral part of the Western area power administration anemometer loan program is employed for analysis in this paper. This data contains average wind speed and the direction in Beloit for the period 2003-2004. The data was originally made available by Wind Powering America, a DOE Office of Energy Efficiency & Renewable Energy (EERE) program. For illustration, the wind speed profile (in hours) of Beloit is shown in Figure 3.

Two data sets are used in this paper for analysis.

- Mins data: in this data set, wind speed is recorded for every 10 mins.
- Hours data: in this data set, wind speed is recorded for every one hour.

In this paper, single-step prediction and six-step ahead prediction were performed on the two data sets separately (six-step ahead prediction of mins data refers to the same duration of single-step prediction of hours data). Comparative analysis is performed with the existing methods for the same data sets to highlight the advantage of the proposed hybrid approach.

### 3.2. Evaluation of Forecast Performance

The indices employed to evaluate the wind forecasting performance are mean absolute error (MAE) and mean absolute percent error (MAPE). Smaller values of these indices imply better forecasting performance.

The indices are defined as follows:

(8) $$\begin{array}{c} \text{MAE}=\frac{1}{T}\sum_{t=1}^{T}\left|s\left(t\right)-\hat{s}\left(t\right)\right|\\ \text{MAPE}=\frac{1}{T}\sum_{t=1}^{T}\frac{\left|s\left(t\right)-\hat{s}\left(t\right)\right|}{s\left(t\right)}\times 100\%, \end{array}$$

where *s*(*t*) is the actual observation value for a time period *t* and $\hat{s}(t)$ is the forecast value for the same time period. The MAE reveals the average variance between the true value and forecast value whereas MAPE has a good sensitivity to small changes in data.

### 3.3. Performance Analysis

In this subsection, forecasting of both mins data and hours data is performed with the proposed hybrid model. Further, a comparative analysis is also performed with EMD-AR and EMD-LSSVM.

**Figure 4:**
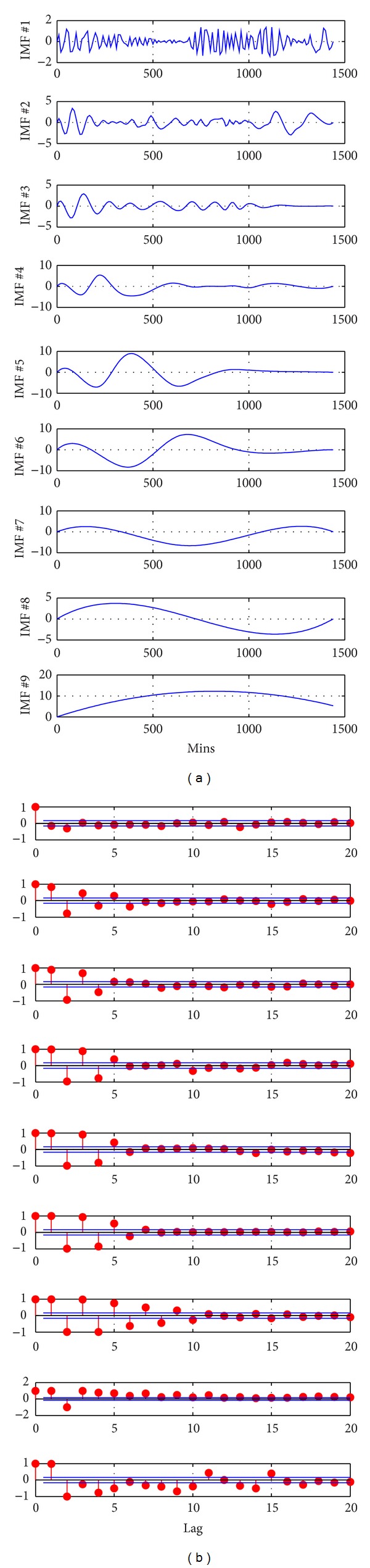
(a) The decomposition of the wind speed profile for Beloit by EMD (b) PACF of respective IMF.

In Stage 1 of the proposed hybrid approach, the wind speed data (mins data) is decomposed into nine IMFs with EMD. Further, PACF for each IMF is computed independently. IMFs and the corresponding PACF are shown in Figure 4. Based on the obtained PACF, LS-SVM is selected for prediction of first two IMFs (IMF-1 and IMF-2) and for the rest seven IMFs; AR model with Kalman filter is selected in Stage 2. Using trail-and-error method, the parameters of LS-SVM are initialized as *C* = 100, *σ* = 50, and *N* = 1000. Based on PACF, second order was identified for AR model. In Stage 3, aggregation of all the IMFs prediction is performed to obtain the final forecasting results.

**Figure 5:**
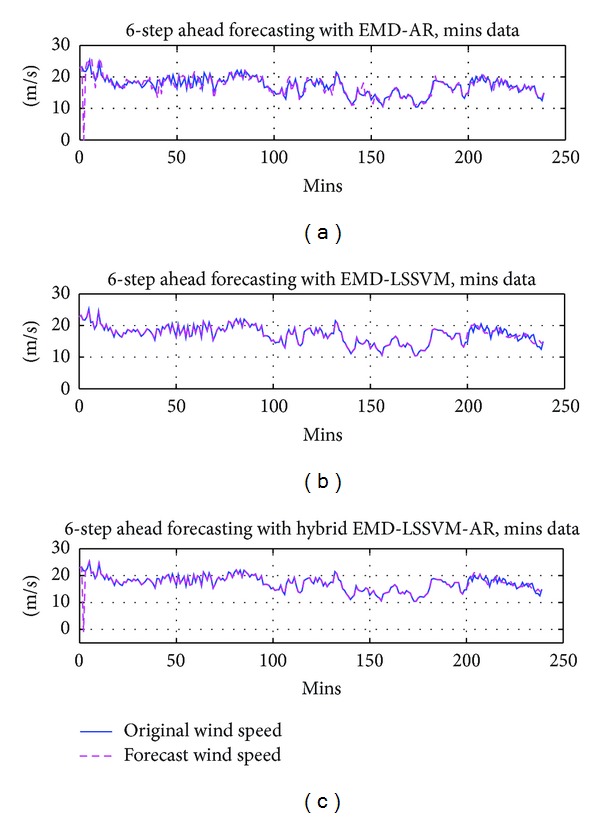
Performance analysis for 6-step ahead forecasting (one hour ahead forecasting) for mins data (a) EMD-AR; (b) EMD-LSSVM; (c) EMD-LSSVM-AR.

For hourly forecasting with mins data, six-step ahead prediction is performed. Results obtained for six-step prediction and single-step prediction with the proposed method along with the existing methods are tabulated in Table 1. The proposed method has an MAE of 0.42 for six-step ahead forecasting, whereas for the existing methods EMD-AR and EMD-LSSVM, MAE obtained was 0.48 and 0.52, respectively. Single-step prediction is performed with hours data for one hour ahead forecasting. The procedure employed for performing the prediction of IMFs is similar to six-step prediction procedure. With the proposed method for single-step prediction, MAE of 0.016 was obtained. With EMD-AR and EMD-LSSVM, MAE obtained was 5.8 and 4.8, respectively. For illustration, the forecasting results for all the three methods for six-step ahead prediction are shown in Figure 5.

**Figure 6:**
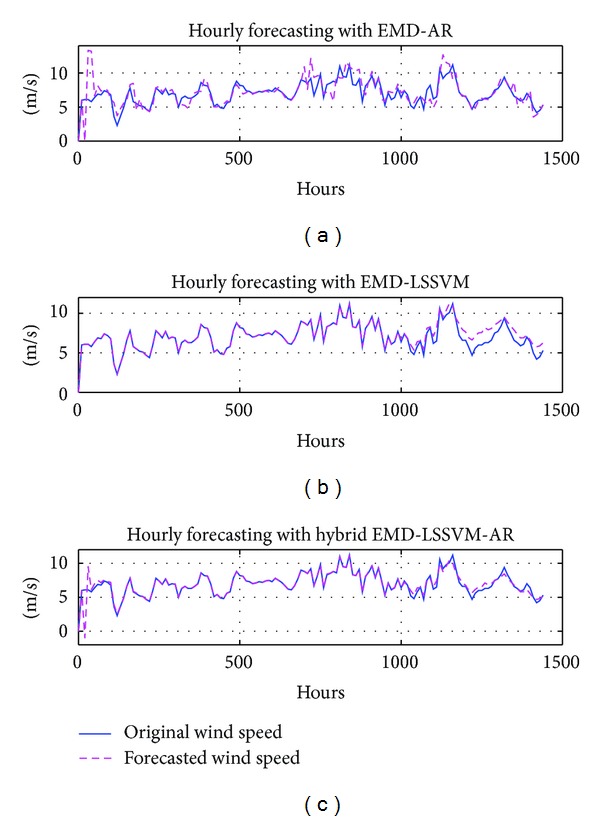
Performance analysis for hourly forecasting (a) EMD-AR (b); EMD-LSSVM; (c) EMD-LSSVM-AR.

To highlight the robustness of the proposed method, six-step ahead prediction with the proposed method is performed. Results obtained are tabulated in Table 1. Results show that the proposed method provides better forecasting compared to the existing methods. For illustration, six-step ahead forecasting results with hours data for all three methods are shown in Figure 6.

## 4. Conclusions

In this paper, a hybrid approach that is a combination of EMD, LS-SVM, and AR model-Kalman filter is developed for wind speed forecasting. The data was first decomposed into IMFs based on the PACF, and then multistep prediction was performed with LS-SVM for some IMFs and AR-Kalman filter for the rest of IMFs. With the proposed method, six-step ahead forecasting for both mins data and hours data was performed. A comparative analysis with existing methods EMD-AR and EMD-LSSVM highlights the advantages of the proposed method. Results show that the proposed hybrid approach provides better forecasting compared to the existing approaches.

## Figures and Tables

**Table 1:** Hourly forecasting performance analysis.

Method	Model errors	Mins data		Hours data	
		1 step	6 steps	1 step	6 steps
EMD-AR [23]	MAE (m/s)	0.022	0.43	0.02	0.48
	MAPE (%)	6.2	31.36	5.8	34.19
EMD-LSSVM [22]	MAE (m/s)	0.018	0.49	0.019	0.52
	MAPE (%)	4.6	34.45	4.8	37.49
EMD-LSSVM-AR	MAE (m/s)	0.012	0.39	0.016	0.42
	MAPE (%)	2.8	28.25	3.2	31.19

## Nomenclature

*s*(*t*): Signal
*v*(*t*): Lower envelope
**w**_*t*_: AR coefficients at instant *t*
${\hat{w}}_{t}$: Estimated AR coefficients
*e*_*t*_: Prediction error
**Q**: Covariance of state noise
**s**: Input vector in LS-SVM training set
*ω*: LS-SVM weights vector
*b*: Bias
*α*: Lagrangian multipliers
*K*(·, ·): Kernel function
*y*: Output of the corresponding **s** in LS-SVM
*u*(*t*): Upper envelope
*m*(*t*): Mean of the envelope
*ϵ*_*n*_ and *η*_*n*_: State noise and measurement noise
**K**_*t*_: Kalman gain vector
**P**_*t*_: Error covariance matrix
*R*: Variance of measurement noise
*N*: Number of training samples
*φ*(·): Nonlinear mapping
*C*: Regularization constant
*ξ*: Estimation error with LS-SVM
$\hat{y}$: Predicted output with LS-SVM.

## References

[1] Szarka J (2006). Wind power, policy learning and paradigm change. *Energy Policy*.

[2] Akpinar S, Akpinar EK (2009). Estimation of wind energy potential using finite mixture distribution models. *Energy Conversion and Management*.

[3] Kim B, Lee J, Jang J, Han D, Kim K-H (2011). Prediction on the seasonal behavior of hydrogen sulfide using a neural network model. *The Scientific World Journal*.

[4] Shiu A, Lam P-L (2004). Electricity consumption and economic growth in China. *Energy Policy*.

[5] Kavasseri RG, Seetharaman K (2009). Day-ahead wind speed forecasting using f-ARIMA models. *Renewable Energy*.

[6] Lizuma L, Avotniece Z, Rupainis S, Teilans A (2013). Assessment of the present and future offshore wind power potential: a case study in a target territory of the Baltic Sea near
the Latvian Coast. *The Scientific World Journal*.

[7] Mohandes MA, Halawani TO, Rehman S, Hussain AA (2004). Support vector machines for wind speed prediction. *Renewable Energy*.

[8] Parrore TJ, Miller RG (1985). Generalized exponential Markov and model output statistics: a comparative verification. *Monthly Weather Review*.

[9] Erdem E, Shi J (2011). ARMA based approaches for forecasting the tuple of wind speed and direction. *Applied Energy*.

[10] Dong Y, Guo Z, Wang J, Lu H (2010). The forecasting procedure for long-term wind speed in the Zhangye area. *Mathematical Problems in Engineering*.

[11] Liu H, Tian H-Q, Li Y-F (2012). Comparison of two new ARIMA-ANN and ARIMA-Kalman hybrid methods for wind speed prediction. *Applied Energy*.

[12] Louka P, Galanis G, Siebert N (2008). Improvements in wind speed forecasts for wind power prediction purposes using Kalman filtering. *Journal of Wind Engineering and Industrial Aerodynamics*.

[13] Veluvolu KC, Ang WT (2010). Estimation and filtering of physiological tremor for real-time compensation in surgical robotics applications. *International Journal of Medical Robotics and Computer Assisted Surgery*.

[14] Veluvolu KC, Latt WT, Ang WT (2010). Double adaptive bandlimited multiple Fourier linear combiner for real-time estimation/filtering of physiological tremor. *Biomedical Signal Processing and Control*.

[15] Latt WT, Veluvolu KC, Ang WT (2011). Drift-free position estimation of periodic or quasi-periodic motion using inertial sensors. *Sensors*.

[16] Zhang W, Wu J, Wang J, Zhao W, Shen L (2012). Performance analysis of four modified approaches for wind speed prediction. *Applied Energy*.

[17] Bilgili M, Sahin B, Yasar A (2007). Application of artificial neural networks for the wind speed prediction of target station using reference stations data. *Renewable Energy*.

[18] Cadenas E, Rivera W (2009). Short term wind speed forecasting in La Venta, Oaxaca, México, using artificial neural networks. *Renewable Energy*.

[19] Beyer H, Degener T, Hausmann J, Hoffmann M, Rujan P Short term wind speed and power output of a wind turbine with neural networks.

[20] Damousis IG, Alexiadis MC, Theocharis JB, Dokopoulos PS (2004). A fuzzy model for wind speed prediction and power generation in wind parks using spatial correlation. *IEEE Transactions on Energy Conversion*.

[21] Guo Z, Zhao W, Lu H, Wang J (2012). Multi-step forecasting for wind speed using a modified EMD-based artificial neural network model. *Renewable Energy*.

[22] Hu J, Wang J, Zeng G (2013). A hybrid forecasting approach applied to wind speed time series. *Renewable Energy*.

[23] Li R, Wang Y Short-term wind speed forecasting for wind farm based on empirical mode decomposition.

[24] Huang NE, Shen Z, Long SR (1998). The empirical mode decomposition and the Hubert spectrum for nonlinear and non-stationary time series analysis. *Proceedings of the Royal Society A*.

[25] Dätig M, Schlurmann T (2004). Performance and limitations of the Hilbert-Huang transformation (HHT) with an application to irregular water waves. *Ocean Engineering*.

[26] Haykin S (2001). *Adaptive Filter Theory*.

[27] Suykens JAK, Vandewalle J, De Moor B (2001). Optimal control by least squares support vector machines. *Neural Networks*.

[28] Bouzgou H, Benoudjit N (2011). Multiple architecture system for wind speed prediction. *Applied Energy*.

